# 1-(2-Cyano­eth­yl)-1*H*-imidazole-4,5-dicarbonitrile

**DOI:** 10.1107/S1600536812036252

**Published:** 2012-08-25

**Authors:** Hong Yu, Ke-Wei Lei

**Affiliations:** a State Key Laboratory Base of Novel Functional Materials and Preparation Science, Institute of Solid Materials Chemistry, Faculty of Materials Science and Chemical Engineering, Ningbo University, Ningbo 315211, People’s Republic of China

## Abstract

In the title tricyano­nitrile compound, C_8_H_5_N_5_, the *N*-substituted cyano­ethyl group is offset to the imidazole ring [dihedral angle = 75.41 (15)°].

## Related literature

For background to the application of imidazole compounds as ligands, see: Li *et al.* (1955 ▶). For the significance of N atoms in metal complex chemistry, see: Fujita *et al.* (1994 ▶). For examples of some imidazole complexes, see: Martin & Edsall (1958 ▶).
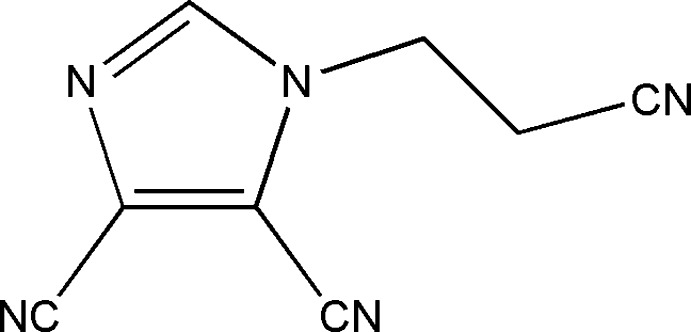


## Experimental

### 

#### Crystal data

C_8_H_5_N_5_
*M* *_r_* = 171.17
Triclinic, 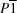

*a* = 6.4831 (6) Å
*b* = 6.7538 (6) Å
*c* = 10.4040 (11) Å
α = 77.865 (9)°
β = 84.297 (8)°
γ = 74.499 (8)°
*V* = 428.71 (7) Å^3^
*Z* = 2
Mo *K*α radiation
μ = 0.09 mm^−1^
*T* = 293 K
0.43 × 0.25 × 0.16 mm

#### Data collection

Rigaku R-AXIS RAPID CCD diffractometer
Absorption correction: multi-scan (*ABSCOR*; Higashi, 1995 ▶) *T*_min_ = 0.973, *T*_max_ = 0.986
3805 measured reflections
2265 independent reflections
1439 reflections with *I* > 2σ(*I*)
*R*_int_ = 0.020

#### Refinement

*R*[*F* ^2^ > 2σ(*F* ^2^)] = 0.047
*wR*(*F* ^2^) = 0.124
*S* = 1.09
2265 reflections
118 parameters
H-atom parameters constrained
Δρ_max_ = 0.13 e Å^−3^
Δρ_min_ = −0.21 e Å^−3^

### 

Data collection: *RAPID-AUTO* (Rigaku, 1998 ▶); cell refinement: *RAPID-AUTO*; data reduction: *CrystalRED* (Rigaku/MSC, 2004 ▶); program(s) used to solve structure: *SHELXS97* (Sheldrick, 2008 ▶); program(s) used to refine structure: *SHELXL97* (Sheldrick, 2008 ▶); molecular graphics: *SHELXTL* (Sheldrick, 2008 ▶); software used to prepare material for publication: *SHELXL97*.

## Supplementary Material

Crystal structure: contains datablock(s) global, I. DOI: 10.1107/S1600536812036252/zs2228sup1.cif

Structure factors: contains datablock(s) I. DOI: 10.1107/S1600536812036252/zs2228Isup2.hkl

Supplementary material file. DOI: 10.1107/S1600536812036252/zs2228Isup3.cml

Additional supplementary materials:  crystallographic information; 3D view; checkCIF report

## Figures and Tables

**Fig. 1.**
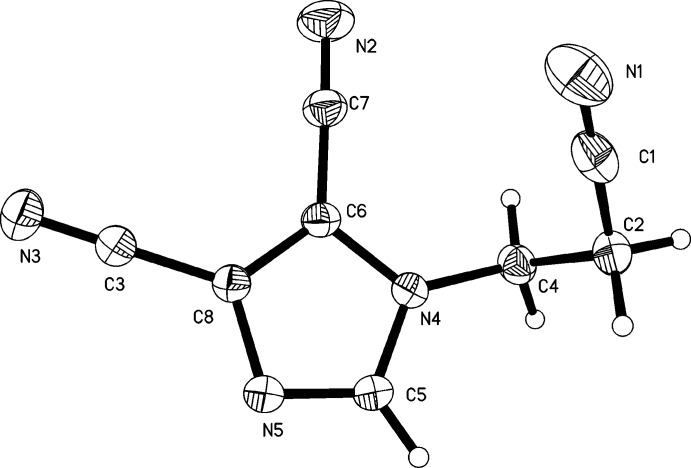
The structure of the title compound, showing 30% probability displacement ellipsoids and the atom-numbering scheme.

## supplementary crystallographic information

### Comment

Imidazole ligands have been used with remarkable success in coordination chemistry over past decades (Li *et al.*, 1955). Some of the reasons are for this are that the N atom plays an important role in the formation of metal complexes (Fujita *et al.*, 1994), and that imidazole complexes show conjugate acid-base properties and good complexation coordination performance in the solid state. Some examples of imidazole complexes have been reported (Martin & Edsall, 1958). Here we report on a new imidazole compound, the polycyano-substituted imidazole, the title compound C_8_H_5_N_5_.

In the molecular structure of this compound (Fig. 1). The bond lengths and bond angles are within normal ranges. The *N*-bound cyanoethyl side chain is offset to the imidazole ring [torsion angles C5—N4—C4—C2 and N4—C4—C2—C1, -78.0 (2) and -61.4 (2)° respectively]. The dihedral angle between the cyanoethyl group defined by atoms N1—C1—C2—C4 and the imidazole ring is 75.41 (15)°]

### Experimental

A mixture of 4,5-dicyanoimidazole (1.18 g, 10 mmol) and powdered potassium hydroxide (100 mg) (as a catalyst) in acrylonitrile (20 ml) was heated at 130 °C for 3 hr in a sealed tube, after which the solution was evaporated to dryness. The crude product obtained was recrystallized twice from acetone to give a pure blue product. Yield: 89.7%. Anal: Calcd. for C_8_H_5_N_5_: C, 56.1; H, 2.9; N, 40.9%: Found: C, 56.15; H, 2.96; N, 40.89%.

### Refinement

All H atoms were placed in geometrically idealized positions and constrained to ride on their parent atoms [C—H = 0.93 Å (aromatic) and 0.97 Å (methylene)], with *U*_iso_(H) = 1.2*U*_eq_(C).

### Crystal data

**Table:**

C_8_H_5_N_5_	*Z* = 2
*M**_r_* = 171.17	*F*(000) = 176
Triclinic, *P*1	*D*_x_ = 1.326 Mg m^−^^3^
Hall symbol: -P 1	Mo *K*α radiation, λ = 0.71073 Å
*a* = 6.4831 (6) Å	Cell parameters from 1220 reflections
*b* = 6.7538 (6) Å	θ = 3.2–29.0°
*c* = 10.4040 (11) Å	µ = 0.09 mm^−^^1^
α = 77.865 (9)°	*T* = 293 K
β = 84.297 (8)°	Block, blue
γ = 74.499 (8)°	0.43 × 0.25 × 0.16 mm
*V* = 428.71 (7) Å^3^	

### Data collection

**Table:**

Rigaku R-AXIS RAPID CCD diffractometer	2265 independent reflections
Radiation source: SuperNova (Mo) X-ray Source	1439 reflections with *I* > 2σ(*I*)
Mirror monochromator	*R*_int_ = 0.020
Detector resolution: 16.1623 pixels mm^-1^	θ_max_ = 29.0°, θ_min_ = 3.2°
ω scans	*h* = −8→8
Absorption correction: multi-scan (*ABSCOR*; Higashi, 1995)	*k* = −9→9
*T*_min_ = 0.973, *T*_max_ = 0.986	*l* = −13→14
3805 measured reflections	

### Refinement

**Table:**

Refinement on *F*^2^	Primary atom site location: structure-invariant direct methods
Least-squares matrix: full	Secondary atom site location: difference Fourier map
*R*[*F*^2^ > 2σ(*F*^2^)] = 0.047	Hydrogen site location: inferred from neighbouring sites
*wR*(*F*^2^) = 0.124	H-atom parameters constrained
*S* = 1.09	*w* = 1/[σ^2^(*F*_o_^2^) + (0.0428*P*)^2^ + 0.0566*P*] where *P* = (*F*_o_^2^ + 2*F*_c_^2^)/3
2265 reflections	(Δ/σ)_max_ < 0.001
118 parameters	Δρ_max_ = 0.13 e Å^−^^3^
0 restraints	Δρ_min_ = −0.21 e Å^−^^3^

### Special details

**Table:**

Geometry. All e.s.d.'s (except the e.s.d. in the dihedral angle between two l.s. planes) are estimated using the full covariance matrix. The cell e.s.d.'s are taken into account individually in the estimation of e.s.d.'s in distances, angles and torsion angles; correlations between e.s.d.'s in cell parameters are only used when they are defined by crystal symmetry. An approximate (isotropic) treatment of cell e.s.d.'s is used for estimating e.s.d.'s involving l.s. planes.
Refinement. Refinement of *F*^2^ against ALL reflections. The weighted *R*-factor *wR* and goodness of fit *S* are based on *F*^2^, conventional *R*-factors *R* are based on *F*, with *F* set to zero for negative *F*^2^. The threshold expression of *F*^2^ > σ(*F*^2^) is used only for calculating *R*-factors(gt) *etc*. and is not relevant to the choice of reflections for refinement. *R*-factors based on *F*^2^ are statistically about twice as large as those based on *F*, and *R*- factors based on ALL data will be even larger.

### Fractional atomic coordinates and isotropic or equivalent isotropic displacement parameters (Å^2^)

**Table:**

	*x*	*y*	*z*	*U*_iso_*/*U*_eq_	
N4	0.25841 (19)	0.19385 (19)	0.19288 (12)	0.0356 (3)	
N5	0.5564 (2)	0.2497 (2)	0.08299 (14)	0.0438 (4)	
C8	0.5883 (2)	0.0426 (2)	0.13842 (15)	0.0367 (4)	
C7	0.3704 (3)	−0.1861 (3)	0.28068 (17)	0.0462 (4)	
C6	0.4074 (2)	0.0040 (2)	0.20743 (15)	0.0353 (4)	
C5	0.3567 (2)	0.3342 (2)	0.11807 (16)	0.0420 (4)	
H5A	0.2902	0.4759	0.0938	0.050*	
N3	0.9384 (2)	−0.2328 (3)	0.10572 (16)	0.0632 (5)	
C4	0.0347 (2)	0.2341 (3)	0.24210 (16)	0.0425 (4)	
H4A	−0.0060	0.1025	0.2662	0.051*	
H4B	−0.0557	0.3222	0.1723	0.051*	
C3	0.7842 (3)	−0.1089 (3)	0.12134 (16)	0.0430 (4)	
C2	−0.0044 (3)	0.3398 (3)	0.36024 (17)	0.0502 (5)	
H2A	0.0338	0.4727	0.3357	0.060*	
H2B	−0.1557	0.3685	0.3862	0.060*	
N2	0.3452 (3)	−0.3409 (3)	0.3388 (2)	0.0740 (6)	
C1	0.1183 (3)	0.2129 (3)	0.47147 (19)	0.0534 (5)	
N1	0.2152 (3)	0.1117 (3)	0.55746 (19)	0.0798 (6)	

### Atomic displacement parameters (Å^2^)

**Table:**

	*U*^11^	*U*^22^	*U*^33^	*U*^12^	*U*^13^	*U*^23^
N4	0.0358 (7)	0.0354 (7)	0.0338 (7)	−0.0091 (5)	0.0008 (5)	−0.0038 (5)
N5	0.0430 (7)	0.0361 (7)	0.0470 (8)	−0.0091 (6)	0.0055 (6)	−0.0008 (6)
C8	0.0379 (8)	0.0356 (8)	0.0347 (8)	−0.0078 (6)	−0.0010 (6)	−0.0049 (7)
C7	0.0486 (10)	0.0386 (9)	0.0500 (10)	−0.0131 (8)	−0.0032 (8)	−0.0023 (8)
C6	0.0409 (8)	0.0323 (8)	0.0324 (8)	−0.0096 (6)	−0.0032 (6)	−0.0040 (6)
C5	0.0433 (9)	0.0322 (8)	0.0452 (9)	−0.0077 (7)	0.0030 (7)	−0.0004 (7)
N3	0.0538 (9)	0.0568 (10)	0.0660 (11)	0.0044 (8)	0.0037 (8)	−0.0100 (8)
C4	0.0348 (8)	0.0482 (9)	0.0436 (10)	−0.0118 (7)	0.0015 (7)	−0.0067 (8)
C3	0.0430 (9)	0.0419 (9)	0.0407 (9)	−0.0082 (7)	0.0011 (7)	−0.0053 (7)
C2	0.0505 (10)	0.0462 (10)	0.0514 (11)	−0.0108 (8)	0.0123 (8)	−0.0124 (8)
N2	0.0836 (13)	0.0471 (10)	0.0879 (14)	−0.0269 (9)	−0.0008 (10)	0.0065 (9)
C1	0.0591 (12)	0.0660 (13)	0.0442 (11)	−0.0288 (10)	0.0112 (9)	−0.0204 (10)
N1	0.0850 (14)	0.1093 (17)	0.0516 (11)	−0.0391 (12)	−0.0047 (10)	−0.0104 (11)

### Geometric parameters (Å, º)

**Table:**

N4—C5	1.3509 (19)	C5—H5A	0.9300
N4—C6	1.3729 (18)	N3—C3	1.142 (2)
N4—C4	1.4630 (19)	C4—C2	1.515 (2)
N5—C5	1.3172 (19)	C4—H4A	0.9700
N5—C8	1.3641 (19)	C4—H4B	0.9700
C8—C6	1.370 (2)	C2—C1	1.452 (3)
C8—C3	1.424 (2)	C2—H2A	0.9700
C7—N2	1.137 (2)	C2—H2B	0.9700
C7—C6	1.416 (2)	C1—N1	1.135 (2)
			
C5—N4—C6	106.18 (12)	N4—C4—C2	112.63 (13)
C5—N4—C4	126.80 (13)	N4—C4—H4A	109.1
C6—N4—C4	126.95 (13)	C2—C4—H4A	109.1
C5—N5—C8	104.35 (13)	N4—C4—H4B	109.1
N5—C8—C6	110.83 (13)	C2—C4—H4B	109.1
N5—C8—C3	122.98 (14)	H4A—C4—H4B	107.8
C6—C8—C3	126.16 (14)	N3—C3—C8	178.15 (19)
N2—C7—C6	178.5 (2)	C1—C2—C4	112.42 (15)
C8—C6—N4	105.61 (13)	C1—C2—H2A	109.1
C8—C6—C7	129.79 (15)	C4—C2—H2A	109.1
N4—C6—C7	124.59 (14)	C1—C2—H2B	109.1
N5—C5—N4	113.02 (14)	C4—C2—H2B	109.1
N5—C5—H5A	123.5	H2A—C2—H2B	107.9
N4—C5—H5A	123.5	N1—C1—C2	179.1 (2)
			
C5—N4—C4—C2	−78.0 (2)	C8—N5—C5—N4	0.08 (17)
C6—N4—C4—C2	105.74 (18)	C5—N5—C8—C3	178.04 (15)
C4—N4—C5—N5	−176.61 (14)	C5—N5—C8—C6	−0.40 (17)
C6—N4—C5—N5	0.25 (18)	C1—C2—C4—N4	−61.4 (2)
C4—N4—C6—C7	−3.8 (2)	N4—C6—C8—N5	0.55 (17)
C4—N4—C6—C8	176.38 (14)	N4—C6—C8—C3	−177.82 (15)
C5—N4—C6—C7	179.31 (15)	C7—C6—C8—N5	−179.22 (16)
C5—N4—C6—C8	−0.48 (16)	C7—C6—C8—C3	2.4 (3)

## References

[bb1] Fujita, M., Kwon, Y. J., Washizu, S. & Ogura, K. (1994). *J. Am. Chem. Soc.* **116**, 1151–1152.

[bb2] Higashi, T. (1995). *ABSCOR* Rigaku Corporation, Tokyo, Japan.

[bb3] Li, N. C., Chu, T. L., Fujii, C. T. & White, J. M. (1955). *J. Am. Chem. Soc.* **77**, 859–861.

[bb4] Martin, R. B. & Edsall, J. T. (1958). *J. Am. Chem. Soc.* **80**, 5033–5035.

[bb5] Rigaku (1998). *RAPID-AUTO* Rigaku Corporation, Tokyo, Japan.

[bb6] Rigaku/MSC (2004). *CrystalRED* Rigaku/MSC Inc., The Woodlands, Texas, USA.

[bb7] Sheldrick, G. M. (2008). *Acta Cryst.* A**64**, 112–122. 10.1107/S0108767307043930 18156677

